# Retrospective analysis of 14 cases of remote epidural hematoma as a postoperative complication after intracranial tumor resection

**DOI:** 10.1186/s12957-015-0754-8

**Published:** 2016-01-06

**Authors:** Jinlu Yu, Hongfa Yang, Dayong Cui, Yunqian Li

**Affiliations:** 1 Department of Neurosurgery, The First Hospital of Jilin University, 71 Xinmin Avenue, Changchun, 130021 People’s Republic of China; 2 Department of Neurosurgery, The Affiliated Hospital of Changchun Chinese Medicine University, Changchun, 130021 China

**Keywords:** Craniocerebral tumor, Surgical resection, Remote, Epidural hematoma

## Abstract

**Background:**

The occurrence of remote epidural hematoma as a postoperative complication after intracranial tumor resection is rare. This study reviewed experiences treating these hematomas and speculated on the causes of this disease. This study reviewed the treatment experience of 14 such cases.

**Methods:**

The 14 patients included 10 males and 4 females, with an age range of 19 to 65 years old. Six cases of tumors occurred in the sellar region, two cases in the lateral ventricle, one case in the fourth ventricle, one case in a cerebellar hemisphere, and four cases in other sites. Among them, five cases were complicated with supratentorial hydrocephalus. The tumors included five cases of meningioma tumors, two cases of pituitary adenomas, three cases of ependymomas, two cases of craniopharyngiomas, one case of astrocytoma, and one case of tuberculosis tumor. For the cases complicated with hydrocephalus, ventricular drainage was provided if needed, and the tumor resection was then performed, with close observation for postoperative changes. If neurological symptoms and disturbance of consciousness occurred, computed tomography (CT) examination was immediately performed. If a remote epidural hematoma was found, the hematoma was evacuated by craniotomy. The patients were followed up after surgery. In the five cases complicated with hydrocephalus, ventricular drainage was first provided for three cases.

**Results:**

All of the 14 cases underwent total tumor resection, and postoperative remote epidural hematoma occurred in all cases, including eight cases on the ipsilateral side and adjacent to the supratentorial operative field; two cases occurred on the contralateral side; two cases occurred on bilateral sides; and two cases occurred in distant areas (with infratentorial surgery, the hematoma occurred on the supratentorial area). Postoperative remote epidural hematoma usually occurred 0.5–5 h after the tumor resection, when the tentorial hernia had already occurred. Following tumor resection and epidural hematoma evacuation, 13 patients were discharged with good recovery, and one patient died.

**Conclusions:**

The reduced intracranial pressure due to the intracranial tumor resection may be the cause of this hematoma. This type of epidural hematoma is acute and often occurs before hernia. Thus, the risk of remote epidural hematoma after intracranial tumor resection needs to be made known. Aggressive hematoma evacuation can often result in satisfactory outcomes for patients.

## Background

Postoperative hemorrhages after craniocerebral tumor resection are not uncommon. They consist mainly of hemorrhage in the surgical cavity due to imprecise hemostasis, subdural hematoma caused by traction on the surface of the cerebral vessels, or contusion and laceration of the brain tissue caused by excessive traction because of the insufficient exposure of the operative field. Epidural hematoma in the surgical area due to the lack of a close subdural stay suture during dural suspension is not uncommon [1–5]. However, the occurrence of remote epidural hematoma after craniocerebral tumor resection is rare. Such remote epidural hematomas can occur in the adjacent area of the ipsilateral side of the surgical area, the contralateral side of the surgical area, the remote areas of the bilateral sides, and even the supratentorial area following infratentorial surgery [6, 7]. These remote epidural hematomas may occur rapidly and appear insidiously, often with large hematoma volumes. When such hematomas have been found, hernias had occurred in most patients, and emergency treatment was required. Currently, the mechanism of the occurrence of this type of remote epidural hematoma is still not fully understood, though it is presumably related to the reduced intracranial pressure caused by the excessive loss of cerebrospinal fluid in the tumor resection [8]. To date, postoperative remote epidural hematoma after intracranial tumor resection has been rarely reported in the literature, and they have been mostly limited to case reports and literature reviews. This study reviewed 14 cases treated in our hospital, covering all types of remote epidural hematoma, and analyses were performed for these clinical data. The significance of this study is that it may contribute to the understanding of these types of remote epidural hematomas.

## Methods

### General information

This study investigated 9178 cases of patients undergoing intracranial tumor resection in the Department of Neurosurgery, First Hospital of Jilin University, from January 2000 to December 2012. Postoperative remote epidural hematomas occurred in 14 cases, including 10 males and four females, with an age range of 19 to 65 years (mean of 42.2 years). The preoperative Karnofsky Performance Status (KPS) scores were 90 in five cases, 80 in eight cases, and 70 in one case. Among the cases, one experienced three surgeries, and one experienced two surgeries. Regarding tumor location, six cases of tumors occurred in the sellar region, two cases in the lateral ventricle, one case in the fourth ventricle, one case in a cerebellar hemisphere, and four cases at other sites. Among them, five cases were complicated with supratentorial hydrocephalus. The tumors included five cases of meningioma tumors, two cases of pituitary adenomas, three cases of ependymomas, two cases of craniopharyngiomas, one case of astrocytoma, and one case of tuberculosis tumor.

### Treatment

#### Tumor resection

The appropriate surgical approach was selected according to the tumor location. For the cases complicated with supratentorial hydrocephalus, especially for infratentorial surgery, ventricular drainage was provided if needed. During tumor resection, the important surrounding nerves and blood vessels were carefully protected. In the case of excessive loss of cerebrospinal fluid after tumor resection with brain tissue collapse, warm saline was added to the surgical cavity during the dural suture, and any air was removed. Holes were drilled at the surgical edge of the skull for stay suture of the dura with precise hemostasis. An epidural drainage tube was placed to avoid excessive drainage. For cases with ventricular drainage, the postoperative cerebrospinal fluid drainage was controlled to prevent excessive drainage.

#### Epidural hematoma evacuation

If postoperative neurological symptoms and disturbance of consciousness occurred after tumor resection, computed tomography (CT) examination was immediately performed to identify the remote epidural hematoma. If epidural hematomas occurred, craniotomy for hematoma evacuation was performed according to the location of the epidural hematoma, as revealed by CT imaging. After clearing the epidural hematoma, stay suture of the dura was conducted with precise hemostasis. For an epidural hematoma occurring bilaterally, bilateral craniotomy for hematoma evacuation was performed. A postoperative drainage tube was placed.

#### Postoperative treatment and follow-up

Conventional symptomatic treatment was provided postoperatively, the same as for other cases of non-remote epidural hematoma. The consciousness status and any physical activity changes were carefully observed. Head CT examination was routinely performed. The patients were discharged after recovery. The postoperative recovery of the patients was followed up with two telephone interviews, with follow-up periods of 6 months and 1 year. Follow-up was performed with KPS scoring.

## Results

### Postoperative results

In the five cases complicated with preoperative hydrocephalus, ventricular drainage was first provided for three cases, including one case of sellar tumor and two cases of infratentorial lesions. All of the 14 cases underwent total tumor resection, and postoperative remote epidural hematoma occurred in all cases, including eight cases on the ipsilateral side and adjacent to the supratentorial operative field, two cases on the contralateral side of the supratentorial operative field, two cases on bilateral sides of the supratentorial operative field, and two cases in distant areas (with infratentorial surgery, the hematoma occurred on the supratentorial area). In the 14 cases of remote epidural hematoma, 11 cases occurred in the vicinity of the sinus, extending to the convex surface. The intervals from tumor resection to the occurrence of postoperative remote epidural hematoma were between 30 min and 5 h in nine cases, 12 h in one case, 18 h in one case, 19 h in one case, 3 days in one case, and 10 days in one case. The Glasgow Coma Scale (GCS) scores before hematoma evacuation and after the occurrence of epidural hematoma were 3–8 in seven cases (tentorial hernia had occurred in all of them), 9–12 in three cases, and 13–15 in four cases.

With respect to the histological grades for the 14 cases involving remote epidural hematoma, the five meningioma cases included three WHO grade II cases and two WHO grade I cases; the two pituitary adenoma cases were both benign; the three ependymoma cases were WHO grade II; the two craniopharyngioma cases were WHO grade I; the one astrocytoma case was WHO grade II; and the one tuberculoma case was benign. Overall, the 14 cases included three cases of benign tumors, four cases of WHO grade I tumors, and seven cases of WHO grade II tumors.

### Treatment results

**Fig. 1:**
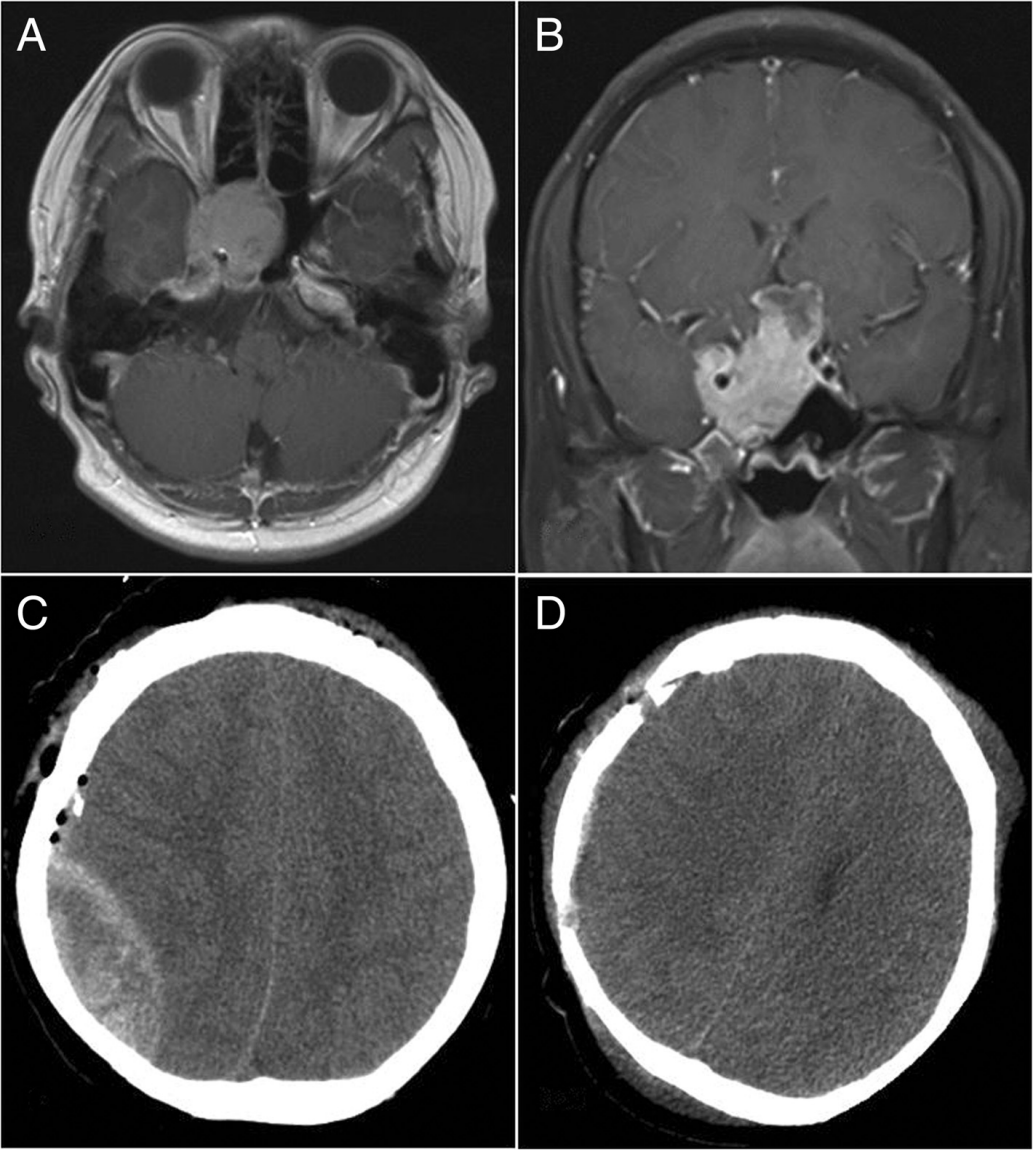
Ipsilateral remote hemorrhage in case 1. **a**, **b** Preoperative-enhanced magnetic resonance imaging (MRI) revealed a sellar tumor and normal ventricular size with no expansion. **c** CT revealed an epidural hematoma behind the surgical field after the tumor resection. **d** CT showed that the hematoma had been cleared

**Fig. 2:**
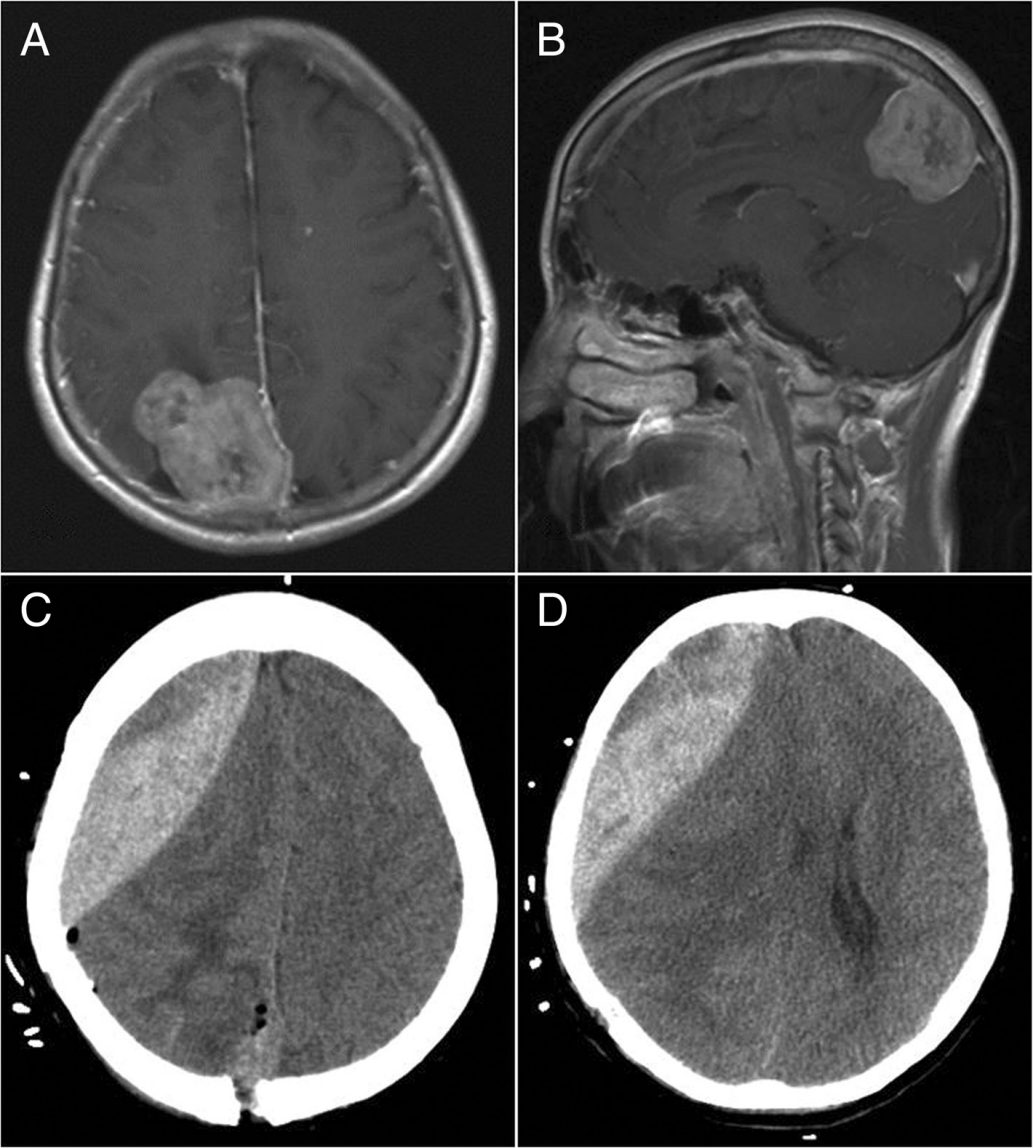
Ipsilateral remote hemorrhage in case 2. **a**, **b** Preoperative-enhanced MRI revealed a meningioma at the top right of the sagittal sinus. **c**, **d** CT showed an epidural hematoma in front of the surgical field after the tumor resection

**Fig. 3:**
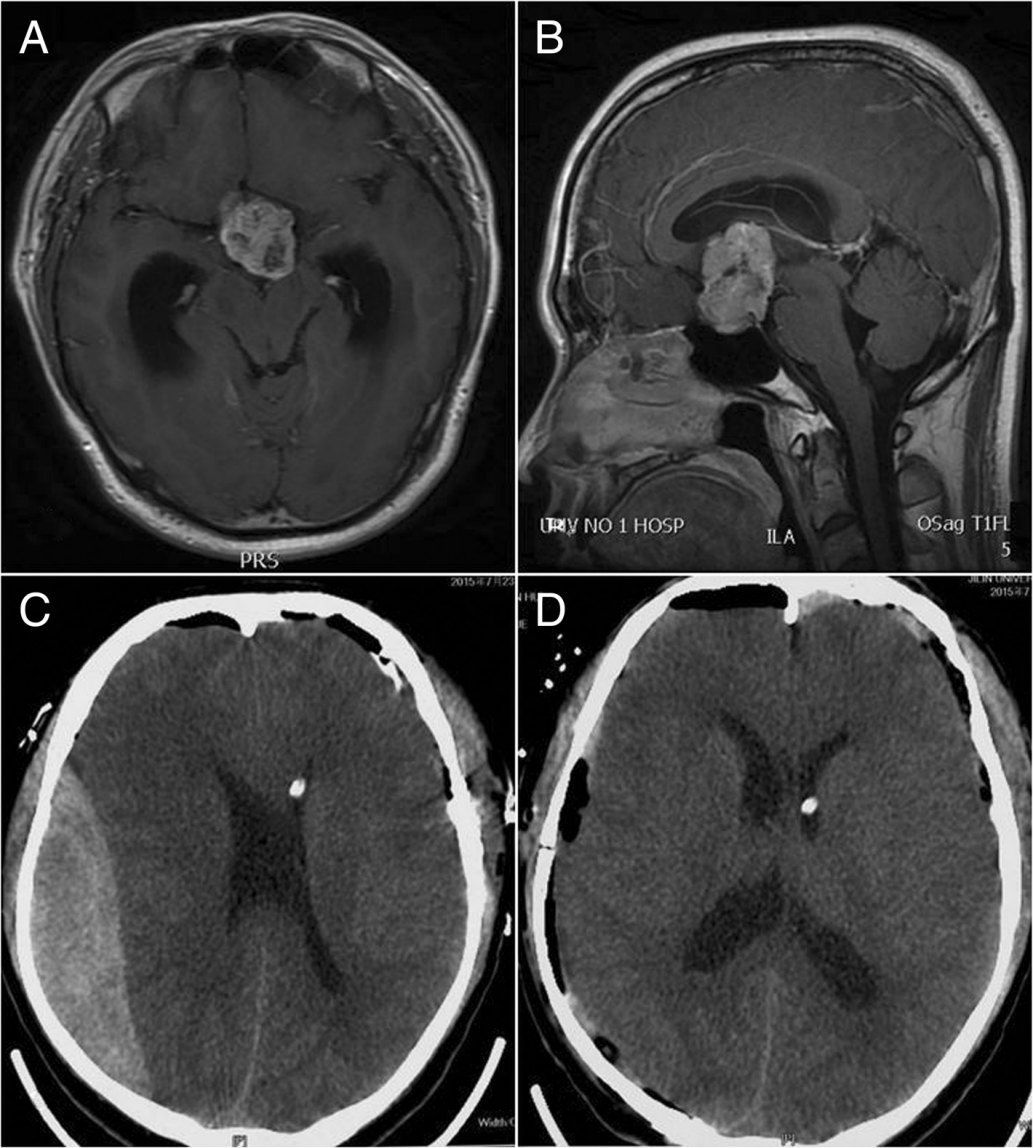
Contralateral remote hemorrhage in case 9. **a**, **b** Preoperative-enhanced MRI revealed a sellar tumor, with ventricular dilatation. **c** CT showed a contralateral epidural hematoma in the surgical field after the tumor resection, with an intraventricular drainage tube. **d** CT showed that the hematoma had been cleared

**Fig. 4:**
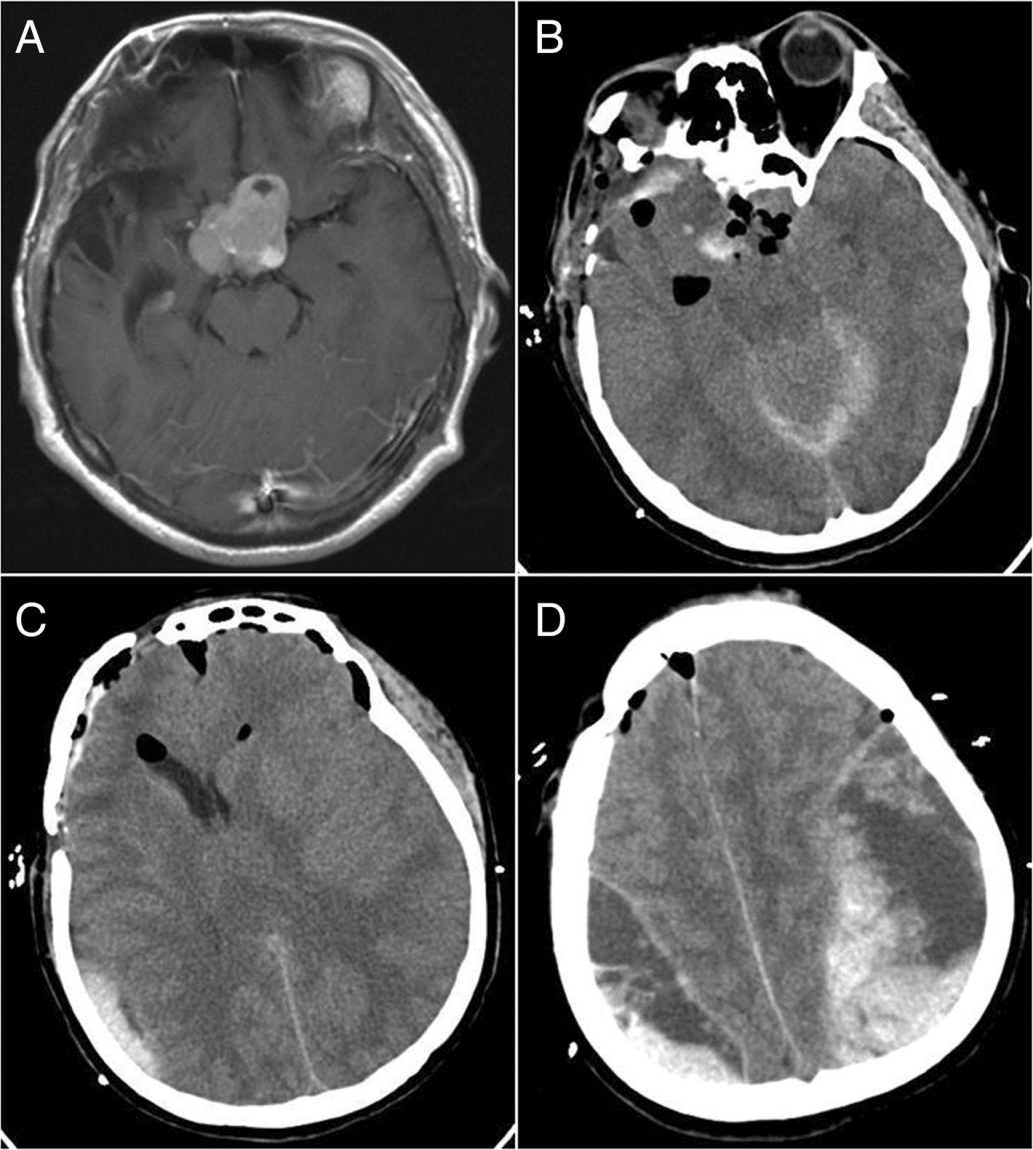
Bilateral remote hemorrhage in case 11. **a** Preoperative-enhanced MRI revealed a sellar tumor. **b** CT showed the tumor resection. **c**, **d** CT revealed epidural hematomas at the tops of bilateral sides

After intracranial tumor resection and epidural hematoma evacuation, 13 patients were discharged with good recovery, and one patient died (the case with epidural hematoma on bilateral sides, case 11). The 13 patients with good postoperative recovery were followed up. The preoperative KPS scores were 90 in five cases, 80 in six cases, 70 in one case, and 50 in one case. The detailed clinical data and treatment results are listed in Table 1. The typical cases are shown in Figs. 1, 2, 3, and 4, while Fig. 4 shows case 11, the case of postoperative death.

**Table 1:** Summary of the clinical data for remote epidural hematomas after intracranial tumor resection

Category	No.	Age	Gender	Preoperative KPS	Surgery history	Tumor location	Hydrocephalus	Type of tumor	Tumor pathology	Surgical approach	Ventricular drainage	Epidural hematoma location	Close to sinus	Interval for secondary surgery	GCS	Cerebral hernia	KPS, 3 months–1 year
Ipsilateral	1	50	Male	80	None	Saddle area	No	Cranio-pharyngioma	WHO grade I	Right frontotemporal	None	Right top	No	3 h	13	No	80
	2	65	Female	90	None	Top right	No	Meningioma	WHO grade II	Top right	None	Right frontal	Yes	5 h	8	Yes	90
	3	36	Male	90	None	Left middle cranial fossa	No	Meningioma	WHO grade II	Left temporal	None	Left occipital	Yes	30 min	11	No	90
	4	25	Male	90	None	Left ventricle	Yes	Ependymoma	WHO grade II	Left frontal	None	Left temporal top	No	12 h	8	Yes	90
	5	45	Female	70	None	Saddle area	No	Pituitary adenoma	Benign	Right frontotemporal	None	Right temporal top	No	4 h	7	Yes	80
	6	42	Male	80	2	Saddle area	No	Pituitary adenoma	Benign	Right frontotemporal	None	Right temporal top	Yes	3 days	13	No	80
	7	36	Male	80	None	Left ventricle	Yes	Ependymoma	WHO grade II	Left frontal	None	Left temporal top	Yes	10 days	12	No	70
	8	34	Female	90	None	Right frontal lobe	No	Astrocytoma	WHO grade II	Right frontal	None	Right occipital top	Yes	5 h	10	No	90
Contralateral	9	34	Male	80	None	Saddle area	Yes	Meningioma	WHO grade I	Left frontotemporal	Yes	Right occipital top	Yes	5 h	13	No	80
	10	56	Male	90	None	By right frontal sagittal sinus	No	Meningioma	WHO grade I	Right frontal	None	Left top	Yes	5 h	13	No	90
Bilateral	11	38	Female	80	3	Saddle area	No	Meningioma	WHO grade II	Right frontotemporal	None	Bilateral top	Yes	1 h	7	Yes	0
	12	56	Male	80	None	Saddle area	No	Cranio-pharyngioma	WHO grade I	Right frontotemporal	None	Bilateral frontal	Yes	2 h	6	Yes	80
Remote	13	55	Male	80	None	Fourth ventricle	Yes	Ependymoma	WHO grade II	Rear middle	Yes	Right temporal top	Yes	18 h	8	Yes	50
	14	19	Male	80	None	Left cerebellar hemisphere	Yes	Tuberculoma	Benign	Rear left occipital paramedian	Yes	Left frontotemporal	Yes	19 h	8	Yes	80

## Discussion

The complication of intracranial hemorrhage after craniotomy is not uncommon. The statistics of 4992 surgical cases surveyed by Kalfas et al. in 1988 showed that postoperative hemorrhage occurred in 40 cases, with a postoperative hemorrhage rate of 0.8 %. Those hemorrhages were mainly intracerebral hematoma (60 %), followed by epidural hematoma (28 %) and subdural hematoma (7.5 %). In the 40 cases, remote hemorrhage from the surgical area occurred in seven cases. Intracranial tumor surgery was the main reason for hemorrhage occurrence, accounting for 56 % of cases, in which meningioma was the main tumor. In the above cases with remote hemorrhage, remote epidural hemorrhage was the most rare, and its cause is not yet well understood [9]. Fukamachi et al. statistically reviewed 1105 cases of epidural hematoma after craniotomy in 1986, including 16 cases of postoperative epidural hematoma, in which 10 cases underwent hematoma evacuation. These 10 cases included four cases involving hematoma in the operative field, five cases involving hematoma in an adjacent region, and one case involving hematoma at a distant site [6]. According to the above statistical analysis of cases with a large sample size, the occurrence of remote epidural hematoma is rare. In this study, a total of 9178 patients undergoing intracranial tumor resection in the Department of Neurosurgery, First Hospital of Jilin University, from January 2000 to December 2012 were reviewed, and postoperative remote epidural hematoma occurred in 14 cases, with an incidence of 0.15 %. These data only presented the incidence of postoperative epidural hematoma after intracranial tumor resection in our center. Therefore, calculations of an accurate incidence rate will require a multi-center study or a larger scale.

The 14 cases of this study were analyzed and showed that these remote epidural hematomas could be classified based on their location. The first type includes hematomas that occur at the adjacent site of the ipsilateral surgical area, but not involving the surgical area; the second type includes hematomas that occur on the contralateral side of the surgical area; the third type includes remote epidural hematomas involving the bilateral sides of the surgical area; and the fourth type includes supratentorial epidural hematomas with infratentorial surgery. In this study, the first type was the most common (eight cases), followed by the contralateral type (two cases), the bilateral type (two cases), and the supratentorial epidural hematoma with infratentorial surgery (two cases). The mechanism of supratentorial remote epidural hematoma after intracranial tumor resection is still not fully elucidated, though a variety of hypotheses have been formulated. Currently, it is commonly accepted that intracranial pressure is reduced after craniotomy due to the substantial loss of cerebrospinal fluid, thereby increasing the dural venous transmural pressure and inducing blood vessel rupture after disorder of the vascular regulation occurs, resulting in epidural hematoma [10–12]. After the bleeding of the torn blood vessels occurs, the dura is stripped off the inner skull plate to form a hematoma, while the pressure effect produced by the hematoma increases the transmural venous pressure, aggravating the bleeding and resulting in hematoma expansion [13, 14]. Some scholars believe that the stretch in the bridging vein due to the brain tissue collapse after the loss of cerebrospinal fluid and the coagulation abnormalities in the patients should also be considered important factors [7, 11]. After reviewing the literature, we found that the occurrence of supratentorial remote epidural hemorrhage after craniotomy was often complicated with either hydrocephalus or hydrocephalus shunts. These patients all showed substantial loss of cerebrospinal fluid after surgery, which supported the above hypothesis [7, 13, 15–17]. In any mechanism, the low intracranial pressure caused by the surgery is the most important triggering factor. In a neurosurgery, the surgical region is usually located in the highest point of the brain so that the ipsilateral dura bears the greatest transmural pressure, with the maximum stretching intensity of the bridging vein. Therefore, remote epidural hematomas most likely occur on the ipsilateral side of the surgical region, which may explain the finding in the present study that, of the 14 cases of hematomas, eight cases occurred on the ipsilateral side and two cases occurred on bilateral sides, resulting in a total of 10 remote epidural hematomas on the ipsilateral side.

According to the above assumption of the postoperative remote epidural hematoma after intracranial tumor resection, the site of supratentorial hemorrhage usually occurs in the vicinity of the sinus because the dural veins on the brain convexity are small, often accompanied with arteries and traveling between two layers of dura; thus, the risk of hemorrhage is relatively low. The anatomical structure of the dura near the venous sinus is complex. After the veins on the brain surface merge to form the thick bridging vein, it transits into the sinus at this location. Additionally, arachnoid granulations in this area easily induce hemorrhage in these structures after the loss of a large amount of cerebrospinal fluid, leading to increased dural vein transmural pressure. This increase tends to occur in younger patients because the adhesion between the dura and the skull in these cases is not very tight [11, 14]. After reviewing the relevant literature, we found that most postoperative supratentorial remote epidural hemorrhages after intracranial surgery were located near the sinus, and the patients were relatively young in age, which supports the above speculation [18, 19]. The average age of the patients in this study was 42 years old, which is also in line with this age characteristic. However, for the first type of epidural hematoma, which occurred on an adjacent site of the ipsilateral side, it should be noted that, in addition to the above assumption, the separation of the surrounding adjacent dura from the inner skull plate during surgery may also induce epidural hematoma because the stay suture of the dura surrounding the surgery area prevents the spread of the hematoma toward the surgical area and thus its extension to distant areas, which may also explain why some of the first types of epidural hematoma did not involve the sinus, such as the example shown in Fig. 2. In addition, two patients in this study had histories of multiple tumor resection, including one case of postoperative remote epidural hematoma on bilateral sides (case 11), whose outcome is believed to be related to the previous multiple surgeries, as the repeated loss of a large amount of cerebrospinal fluid could aggravate a regulation disorder of the intracranial vascular system, thus causing remote bilateral epidural hematoma.

The question of whether an epidural hematoma remote from the surgical region was correlated with the pathological grade of the resected tumor was also analyzed in this study. Among the 14 examined cases, three cases involved benign tumors, four cases involved WHO grade I tumors, and seven cases involved WHO grade II tumors. The benign cases included two cases of pituitary adenoma and one case of tuberculoma (a type of lesion that is not included in the WHO pathological grading of central nervous system tumors). Therefore, this study examined seven cases involving benign and WHO grade I tumors and seven cases involving WHO grade II malignant lesions. Benign and malignant lesions each accounted for half of the included cases; thus, remote epidural hematoma appears to be independent of the resected tumor’s pathological grade. Based on a literature review, numerous similar remote epidural hematomas have been reported after trauma surgery or hydrocephalus surgery, and these hematomas were clearly related to reduce intracranial pressure [11, 12, 20].

Among the 14 cases included in this study, there were five meningioma cases. Thus, the question of whether remote epidural hematoma occurs particularly frequently in meningioma cases is also examined in this study. The current consensus is that such remote epidural hematomas are related to craniotomy-induced decreased intracranial pressure but are not correlated with meningioma [18, 19]. This hypothesis was supported by a 1986 study by Fukamachi et al., who statistically analyzed 1105 cases involving craniotomy and found that remote epidural hematoma mainly occurred in patients with hydrocephalus and/or aneurysm [6]. Recently, in 2015, Chung et al. reported three cases of remote epidural hematoma after brain tumor surgery, none of which involved meningioma; therefore, this type of remote hemorrhage appears to be independent of brain tumor type [21].

These postoperative remote epidural hematomas after intracranial tumor resection occur in the epidural region and show no parenchymal damage except for oppression on the brain tissue; therefore, timely hematoma evacuation can effectively relieve the oppression of the hematoma on the brain tissue. In this study, 13 out of 14 cases achieved good prognoses, with satisfactory results in terms of KPS scores. It should be noted that these epidural hematomas often progresses rapidly. In the present study, seven cases (50 %) exhibited rapid disease progress, and cerebral hernias had occurred by the time the hematomas were found, often within 30 min to 5 h after surgery (nine cases). GCS scores were used to accurately assess the condition of the postoperative remote epidural hematoma when exacerbation occurred. Although the vast majority of remote epidural hematomas in the present study achieved satisfactory results after aggressive treatment, some cases still show poor prognoses. As an example, for the bilateral epidural hematoma that occurred in case 11, the disease condition was more dangerous than that of a unilateral hematoma. Due to the hematoma’s rapid progression, the large bleeding volume, and repeated surgery, even a bilateral hematoma evacuation failed to save the patient’s life. Therefore, extra attention should be paid to remote epidural hematomas occurring on bilateral sides, which might result in poor therapeutic effects.

## Conclusions

In summary, according to the location of the hematoma, postoperative remote epidural hematomas occurring after intracranial tumor resection can be classified as four types, including those at adjacent sites, on the contralateral side, on bilateral sides, and supratentorial epidural hematoma with infratentorial surgery. The reduced intracranial pressure due to the intracranial tumor resection may be the cause of these hematomas. For the first type of epidural hematoma, which occurs adjacent to the surgical area, in addition to the low intracranial pressure, the separation of the dural edge due to the surgery may also be a causal factor. The occurrence of such postoperative epidural hematomas is often acute, and in these cases, cerebral hernias had usually already occurred by the time the hematoma was found. Thus, the risk of remote epidural hematoma after intracranial tumor resection needs to be made known. This study found that aggressive hematoma evacuation could provide patients with satisfactory outcomes.

### Consent

Written informed consent to the publication of this study and its accompanying images was obtained from the included patients.
